# Serum Level of Xanthine Oxidase, Uric Acid, and NADPH Oxidase 1 in Stage I of Multiple Myeloma

**DOI:** 10.31557/APJCP.2020.21.8.2237

**Published:** 2020-08

**Authors:** Maryam Kohsari, Mohammad Hassan Khadem Ansari, Yousef Rasmi

**Affiliations:** *Department of Biochemistry, Faculty of Medicine, Urmia University of Medical Sciences, Urmia, Iran. *

**Keywords:** Multiple myeloma, xanthine oxidase, uric acid, NADPH oxidase 1

## Abstract

**Objective:**

The etiology of multiple myeloma (MM) is not known. Enzymes such as xanthine oxidase (XO) and NADPH oxidase 1 (NOX1) as relevant sources of reactive oxygen species (ROS) production may play a crucial role in the incidence and progress of MM. Uric acid generated by XO has a controversial dual role in both the prevention and promotion of cancer. We conducted a case-control study and selected patients with stage I MM to investigate the status of XO, NOX1, and uric acid in the patients and controls.

**Methods:**

We used a sample of 33 patients with stage I MM and 30 healthy controls. The enzyme-linked immunosorbent assay (ELISA) measured the enzyme concentration of XO and NOX1, and the colorimetric method measured the serum level of uric acid.

**Results:**

Mean serum levels for XO in patients and controls were 6.17±0.83 ng/ml and 4.12±0.57 ng/ml (P<0.001). serum levels of NOX1 were 4.35±1.03 ng/ml in patients and 3.54±0.91 ng/ml in controls (P<0.001). Evaluating the levels of XO and NOX1 in male and female populations showed a significant difference in the male population (NOX1 P=0.002; XO P<0.001) and female population (NOX1 P=0.002; XO P<0.001). Also, a significant correlation was observed between the two enzymes only in the female population (Pearson correlation=0.5; P=0.006). A significant inverse correlation found between albumin and XO (Pearson correlation=-0.7, P<0.001) and NOX1 (Pearson correlation=-0.5, P<0.001). XO was correlated with B2-m (Pearson correlation=0.37, P=0.003). There was no significant difference in uric acid between patients (6.2±1.2 mg/dl) and controls (5.7±1 mg/dl) (P=0.2), and no correlation was found with XO.

**Conclusion:**

The present study indicates the possible role of XO and NOX 1 in the etiology of MM. Although we found no correlation between uric acid and XO, further studies will help clarify the function of uric acid in the pathogenesis of MM.

## Introduction

Multiple myeloma (MM) results from malignancy in plasma cells (Rajkumar and Kumar, 2016). The pathogenesis of MM is complicated, and its exact cause is unknown (Kuku et al., 2005). Oxidative stress arises when the creation of reactive oxygen species (ROS) overwhelms the natural antioxidant defenses (Hoye et al., 2008). ROS are the important signaling mediator that take part in different cell biological activities, including cell signaling, angiogenesis, and differentiation (Rodrigues et al., 2008). Evidence suggests that the ROS increase in malignant cells induces proliferation, motility, and invasion (Burdon, 2005; Hamanaka and Chandel, 2010), although the precise relationship of ROS with this process is not well understood. NADPH oxidase (NOX) and xanthine oxidase (XO) are essential sources of ROS (Li et al., 2013) .

XO is an oxidase and the most circulating form of xanthine oxidoreductase; in humans, XO is ordinarily present as xanthine dehydrogenase that converts into XO in pathologic and ischemic conditions (Houston et al., 1999). The XO catalyzes the conversion of hypoxanthine and xanthine to uric acid, and during the reaction, produces superoxide anion (. O2-) (Boueiz et al., 2008; Nishino et al., 2005). XO can directly stimulate the metabolic pathways of carcinogenesis (Battelli et al., 2016). Evidence suggests the dual role of XO in both oncogenic (Shmarakov and Marchenko, 2009) and preventive (Valko et al., 2006) conditions. In tissues with a high expression of the enzyme such as breasts, ovaries, liver, intestine, and kidneys, malignancy is associated with decreased XO levels. In contrast, in other tissues with low enzyme expression, malignancy is associated with an increased XO expression; also, there is a controversial role of uric acid produced by XO present in the pathogenesis of cancer (Battelli et al., 2016). Uric acid produced by XO has an anti-oxidant activity within the circulation that may be relevant in the prevention of cancer. On the other hand, uric acid may present a pro-oxidant and tumor promoter activity, mainly within the cell, conceivably because of the free radicals derived from its reaction with ROS. The dual role of uric acid has become a controversial topic in cancer research (Ames et al., 1981; Battelli et al., 2018; Neogi et al., 2012) .

The NOX family is a group of transmembrane proteins that transport electron from the NADPH to (O2-) and then to hydrogen peroxidase (H_2_O_2_) (Dupuy et al., 1999). NOX1, an isoform of NOX, plays a well-known role in malignancy progression, angiogenesis, and signaling pathway in cancer development that has raised its significance in the NOX family (Kamata, 2009).

To expand information about the etiology of MM and check the oxidative stress status in patients with stage I MM, we conducted a case-control study to assess the serum level of XO, NOX1, and uric acid in patients with stage I MM and healthy controls.

## Materials and Methods

The study involved 63 participants, including 33 patients with stage I MM (17 males and 16 females) with an average age of 62.11± 6.7 years, and 30 healthy controls (16 males and 14 females) with an average age of 60.2±4.8 years. All the patients newly diagnosed with MM were staged according to the International Staging System (ISS) for MM (Greipp et al., 2005). Only patients with stage I MM were included. None of the patients had received chemotherapy or radiotherapy before sampling. Also, healthy controls were examined for the absence of any underlying disease that might affect the conditions of the test. Blood samples were obtained after overnight fasting. The serum was separated via centrifugation at 300 g for 10 minutes, and then transferred to cryotubes, stored at -70°C, and assayed at the end of the study to avoid inter-assay variability.

*Measurement of XO and NOX1*

The quantitative sandwich enzyme immunoassay method using Bioassay Technology Laboratory (ShanghaiCrystal Day Biotech Co.) was performed to measure the serum level of XO and NOX1, LTD. The standard and samples were pipetted into the wells of a microplate on which a monoclonal antibody specific for XO and NOX1 had been pre-coated; the immobilized antibody bounds any XO and NOX1 present. The microplate was washed, and an enzyme-linked monoclonal antibody specific for XO and NOX1 was added to the wells. After adding the solution substrate, the intensity of the color (which develops in each well in proportion to the amount of XO and NOX1 bound during the initial step) was measured by an ELISA reader (Lab system Multiskan MS, USA) at the wavelength of 450 nm and 630 nm. 

*Measurement of Uric Acid*

The measurement of uric acid serum level was performed using the uric acid assay kit (Pars Azmun, Tehran, Iran); this colorimetric assay was carried out according to the manufacturer’s instructions (Pars Azmun uric acid). The optical density was read at 547 nm with a BT3000 Plus^®^ (Italy).

*Measurement of Albumin*

Serum albumin measurement was performed (Pars Azmun, Tehran, Iran) according to the manufacturer’s instructions. The optical density was read at 578 nm with a BT3000 Plus^®^ (Italy).

*Measurement of β2-microglobulin*

Serum β2-microglobulin measurement (LIAISON^®^, DiaSorin, Italy) was conducted according to the manufacturer’s instructions.

*Statistical analysis*

The statistical assessment was carried out with the SPSS 16.0 software (SPSS Inc, released 2007; SPSS for Windows, Version 16.0. Chicago, USA). First, the Kolmogorov-Smirnov test was performed to check the normality of the data. An independent samples t-test was used to compare the values between the patients with stage I MM and the controls. Pearson’s correlation analysis was used to find associations between the parameters. Moreover, a regression analysis was applied to see the prediction between two test factors. A P-value < 0.05 was considered statistically significant. The results are expressed as mean ± standard deviation. 

## Results

The results of the means of albumin, β2-microglobulin, and uric acid in patients and controls are given in Table 1.

Serum levels of XO and NOX1 were higher in patients than in controls.

**Figure 1:**
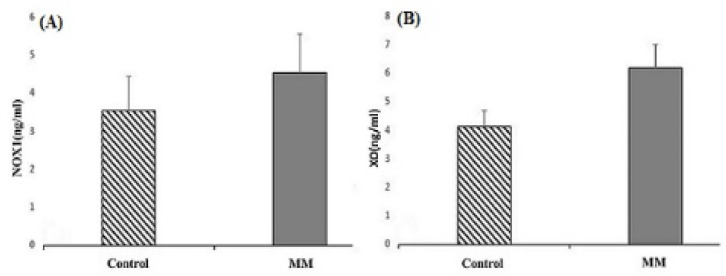
The Mean NOX1 (A) and XO (B) Serum Levels between Patients with MM and Controls

**Figure 2:**
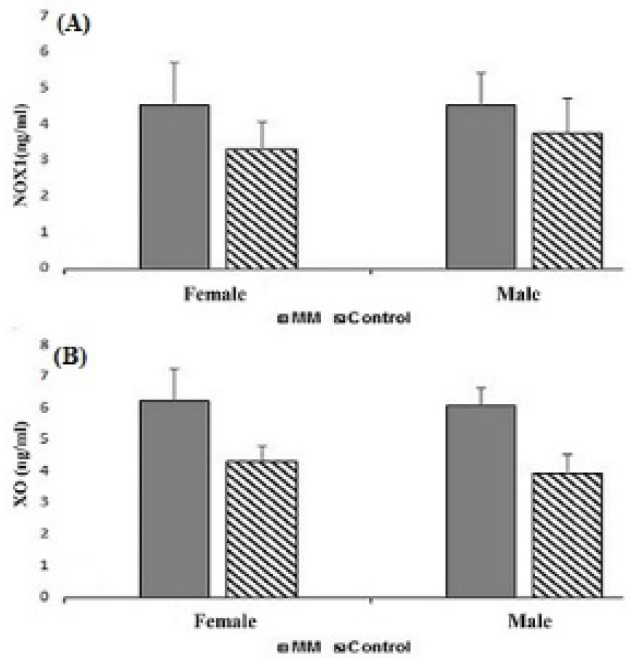
The Mean Levels of NOX1(A) and XO(B) in the Population of Males and Females. There was a significant difference between the mean of NOX1 and XO in the MM patients and controls in both the male and female populations

**Figure 3:**
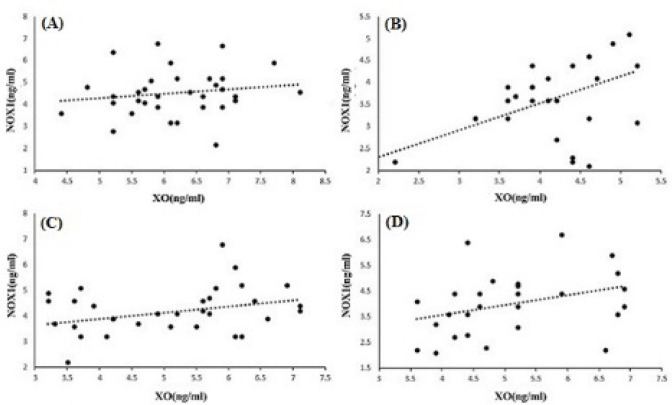
The Correlation between NOX1 and XO in Patients (A), controls (B), population of males (C) and population of females (D). Significant correlations were observed only in the female (MM and controls) population (P=0.006).

**Figure 4:**
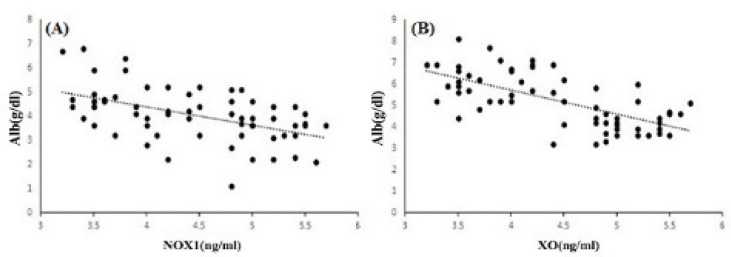
The Significant Inverse Correlation between Serum Levels of Albumin with NOX1 (A) (P<0.001) and XO (B) (P<0.001).

**Figure 5:**
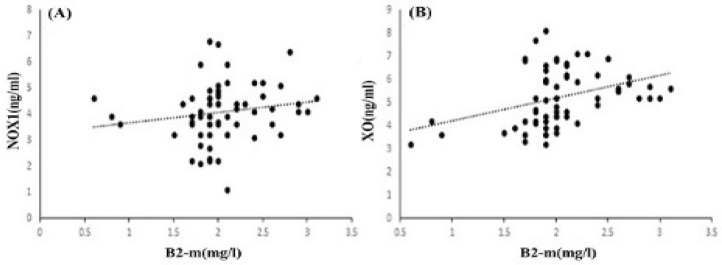
The Correlation between NOX1(A) and XO(B) with β2-m. XO was Significantly Correlated with Serum Levels of β2-m (P=0.003)

The results showed a significantly increased level and a significant difference between the mean of patients and controls for XO. The means of XO were 6.17±0.83 ng/ml in patients and 4.12±0.57 ng/ml in controls (P<0.001). We obtained similar results for NOX1. The means of NOX1 were 4.35±1.03 ng/ml in patients and 3.54±0.91 ng/ml in controls (P<0.001) (Figure 1). In the two studied enzymes in the male and female populations, there was an increase in serum level and a significant difference between the mean of the two sexes. The mean of XO was 6.1±0.56 ng/ml in male patients and 3.95±0.58 ng/ml in male controls (P<0.001). NOX1 in male patients and male controls equaled 4.52±0.92 ng/ml and 3.73±1 ng/ml, respectively (P=0.02). The mean of XO was in female patients (6.25±1 ng/ml) and female controls (4.32±0.5 ng/ml) (P<0.001). NOX1 in female patients and female controls was 4.5±1.18 ng/ml and 3.31±0.77 ng/ml, in that order (P=0.002) (Figure 2). The Pearson correlation test showed a significant correlation between enzymes only in the female population (Pearson correlation=0.5, P=0.006) (Figure 3). Both serum levels of XO and NOX1 had a significant inverse correlation with serum levels of albumin, and results respectively for XO and NOX1 were: Pearson correlation=-0.5, P<0.001, and Pearson correlation=-0.7, P<0.001 (Figure 4). Furthermore, there was a positive correlation between the serum levels of XO and B2-microglobulin (Pearson correlation=0.37, P=0.003) (Figure 5). There was no significant difference between the mean serum levels of uric acid in the patients compared with the controls (Table 1); also, there was no correlation between the serum levels of XO and uric acid.

**Table 1:** The Mean Serum Albumin, β2-Microglobulin, and Uric Acid Levels in MM Patients and Controls

	MM	Controls	P-value
Albumin (g/dl)	3.82±0.4	5/09±0.3	<0.001
β2-microglobulin (mg/l)	2.25±0.4	1.8±0.4	<0.001
Uric acid (mg/dl)	6.2±1.27	5.8±1	0.27

## Discussion

XO is the source of oxygen free radicals in polymorphic, epithelial, endothelial, and connective tissue cells (Chung et al., 1997). Our results demonstrated increased serum levels of XO in patients. Swaminathan and Hatcher showed the role of XO inactivation of the carcinogenesis pathway in bladder cancer (Swaminathan and Hatcher, 1986), and Kono et al., (2012) reported an increased expression in mRNA xanthine dehydrogenase (consequently, XO) along with increased malignancy and decreased survival in the adenocarcinoma of the lung. Because the MM environment (bone marrow) (Edwards et al., 2008) is not part of the enzyme-secreting tissues (Battelli et al., 2016), increased serum XO levels may play an active role in the development of MM. There are also conflicting results about the uric acid produced by XO. The effect of uric acid can be due to its intracellular increase leading to ROS production and stimulation of tumorigenesis (Fini et al., 2012). The literature shows an association between increased uric acid and reduced synthesis of E-cadherin and induction of the epithelial-mesenchymal transition (EMT) process during the progression of renal tubular cancer (Ryu et al., 2013); therefore, increasing uric acid by inducing the differentiation of immunosuppressive IDO+/IL10+ lymphocytes can reduce tumor surveillance (Eisenbacher et al., 2014).

Contrary to these results, high levels of uric acid are associated with a reduced risk of lung and colon cancer and a good prognosis in patients (Dziaman et al., 2014; Horsfall et al., 2014). In our study, although the serum level of uric acid was higher in patients than in controls, the difference was not significant; also, we did not find any correlation between the serum levels of XO and uric acid in patients and controls. A study of head and neck cancer linked uric acid rise to staging and suggested that uric acid could be a prognostic factor (Dhankhar et al., 2011). To the best of our knowledge, for the first time, we tested the serum levels of XO and uric acid in patients with stage I MM. The results of our study demonstrated a significant inverse correlation between XO and serum albumin and a positive correlation with B2-microglobulin; this may indicate the critical role of XO in MM and its association with disease progression and activity. Therefore, further studies and follow up for patients are needed to clarify the relationship between increased levels of XO and uric acid and the effect of uric acid on the disease process. We found observed a correlation between the serum level of NOX1 with XO expression in the population of females, and an inverse correlation with serum albumin.

NOX1 is generated in various normal and malignant cells (Geiszt, 2006). In the present study, we observed increased levels of NOX1 in patients with stage I MM compared with controls. No similar study investigating NOX1 in patients with MM was found. Only the study by Bustany et al., (2016) reported that ROS generated from NOX may have played a role in the imbalance of the redox status in myeloma cells. In terms of other hematologic malignancies, the results of Ready et al. showed that the NOX family affects myeloid cell proliferation and migration by oncogenic tyrosine kinase, and they have been able to purify NOX1 from murine myeloid cells (Reddy et al., 2011). NOX1 stimulates morphogenesis changes in endothelial cells (Kobayashi et al., 2004; Wen et al., 2013), and the ROS produced by NOX1 is associated with increased angiogenesis (Arbiser et al., 2002). Studies support that an increase in ROS generated from NOX1 activates the NF-κB signaling pathway which is associated with the early stages of tumor activity (Kajla et al., 2012). Other studies have emphasized the role of oxidative stress and NOX1 proliferate malignant cells in the early stages of cancer (Arnold et al., 2007; Dreher and Junod, 1996). NOX1 is produced by fibroblast (Suh et al., 1999) and osteoclast (Geiszt, 2006) cells that play a crucial role in the development and survival of myeloma cells in the bone marrow (Mundy, 1998; Mundy et al., 1974). NOX1 seems to be a potential factor in the pathogenesis of MM, and further studies are required to understand its role.

In conclusion, the findings of our study displayed the probable role of XO and NOX1 in the incidence and progress of MM. We found that XO is correlated with B2-m in stage I MM and may have a role in its progress, but further studies are needed to follow up the patients to demonstrate the prognostic value of XO in MM. Also, the inverse correlation of XO and NOX1 with serum albumin in stage I shows the role of these two factors in MM activity. We did not find a significant association between uric acid and XO; however, due to the controversial role of uric acid in the pathogenesis of cancer, further studies can .help clarify its function. Our results may help a better understanding of the etiology of the disease and finding new methods of treatment. Studies on oxidative stress and its causative agents in blood malignancies have begun in recent years, and our results can provide the basis for future studies.
